# Case Report: Primary inferior vena cava leiomyosarcoma with hepatic metastases initially misinterpreted as primary hepatic leiomyosarcoma – ultrasonographic features and diagnostic insights

**DOI:** 10.3389/fonc.2026.1830666

**Published:** 2026-09-03

**Authors:** Yimei Deng, Yu Song, Shasha Shi, Rui Cong

**Affiliations:** Department of Ultrasound, The Second Hospital of Dalian Medical University, Dalian, China

**Keywords:** case report, diagnosis, inferior vena cava, leiomyosarcoma, ultrasonographic features

## Abstract

**Background:**

Leiomyosarcoma of the inferior vena cava is a rare and malignant soft tissue tumor. Diagnosis is often delayed due to non-specific symptoms. Imaging is crucial, yet preoperative differentiation remains challenging.

**Case presentation:**

A 59-year-old woman presented with a two-year history of right upper quadrant pain. Initial ultrasound revealed a large, well-defined, hypoechoic hepatic mass (10.6 × 7.8 cm) with heterogeneous echotexture featuring multiple strip-like hyperechoic regions and irregular anechoic areas. Contrast-enhanced ultrasound showed a malignant enhancement pattern. Chest CT indicated pulmonary metastases. Initial ultrasound diagnosis was primary hepatic leiomyosarcoma. Ultrasound-guided biopsy confirmed leiomyosarcoma. Subsequent targeted comprehensive imaging re-evaluation, including retrospective review of ultrasound cine loops and MRI sequences, demonstrated tumor continuity with the IVC lumen, leading to the final diagnosis of primary inferior vena cava leiomyosarcoma with hepatic extension and pulmonary metastases.

**Conclusions:**

This case demonstrates that targeted ultrasound re-evaluation is valuable for clarifying the tumor–IVC relationship. The sonographic finding of multiple strip-like hyperechoic regions may be a characteristic feature of inferior vena cava leiomyosarcoma. We propose that leveraging imaging similarities between smooth muscle tumors across different anatomical sites (e.g., uterus, peripheral veins) can foster a cross-organ diagnostic mindset. Furthermore, exploring cross-organ, cross-modality transfer learning models could address data scarcity and enhance diagnostic capabilities for rare entities like inferior vena cava leiomyosarcoma.

## Introduction

Leiomyosarcoma of the inferior vena cava (IVC LMS) is a rare malignant tumor, with the IVC being the most common site for primary venous leiomyosarcoma (1, 2). Clinical presentations are often non-specific. Diagnosis relies on histopathology (positive for smooth muscle actin, desmin, and vimentin).

Since its initial description in 1871, IVC LMS has been predominantly documented in case reports and small retrospective series (3). While CT and MRI are primary modalities for characterization and staging, the role of ultrasound is less documented (4, 5). This report presents a case of primary IVC LMS, with a particular focus on the characteristic sonographic features of the tumor, aiming to explore the potential value of ultrasound in the diagnosis of this rare soft tissue sarcoma.

Case description:

A 59-year-old woman presented with worsening right upper quadrant pain radiating to the back, abdominal distension, and diarrhea for two months, following a two-year history of dull discomfort. Laboratory tests showed elevated neuron-specific enolase (NSE) (25.9 ng/mL); other tumor markers were normal. External CT noted a hypodense liver mass and bilateral pulmonary nodules.

**Figure 1:**
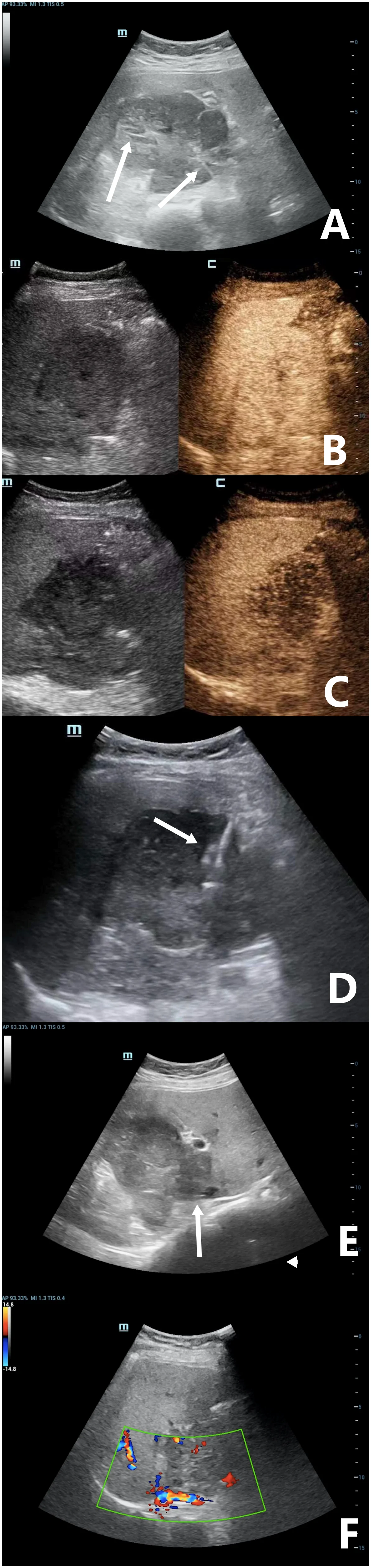
Ultrasound findings of IVC leiomyosarcoma. **(A)** Grayscale image shows a heterogeneous hypoechoic mass with multiple strip-like hyperechoic regions (arrows) and irregular anechoic areas suggesting necrosis. **(B)** Contrast-enhanced ultrasound (CEUS) in arterial phase demonstrates marked peripheral-to-center hyperenhancement. **(C)** CEUS in delayed phase shows progressive washout with persistent hypoechogenicity. **(D)** Ultrasound-guided percutaneous biopsy (arrow) using a 16G needle. **(E)** Retrospective cine-loop review reveals a hypoechoic component within the IVC lumen (arrow), continuous with the hepatic mass, initially overlooked. **(F)** Color Doppler with pulsed wave trace shows IVC luminal narrowing and increased flow velocity, initially misinterpreted as extrinsic compression rather than intraluminal tumor.

Abdominal ultrasound at our institution revealed a 10.6×7.8 cm, well-defined, hypoechoic mass in the right hepatic lobe. It exhibited a heterogenous echotexture with multiple strip-like hyperechoic regions and scattered irregular anechoic areas, suggesting necrosis (**Figure 1A**). Color Doppler showed abundant internal and peripheral flow. Because the lesion exhibited atypical features for common hepatic malignancies, the tumor markers were within normal limits except for a mild elevation in NSE, a biomarker with limited diagnostic specificity, and pulmonary nodules were present, a rare primary sarcoma was considered. Based on the sonographic resemblance of the lesion to uterine smooth muscle tumors, an attending ultrasound physician with 8 years of experience in clinical ultrasonography, who performed and interpreted the examination, initially suspected primary hepatic leiomyosarcoma. Subsequently, she underwent abdominal and thoracic computed tomography (CT) as well as abdominal magnetic resonance imaging (MRI). MRI confirmed a malignant hepatic mass with heterogeneous T2 signal, restricted diffusion, and heterogeneous enhancement, with suspected involvement of the pancreatic head and right adrenal gland. The contrast-enhanced ultrasound (CEUS) examination revealed a malignant enhancement pattern (**Figures 1B, C**). Under ultrasound guidance, a percutaneous biopsy of the hepatic mass was performed using a 16G biopsy needle (**Figure 1D**). Two tissue cores, each approximately 2.0 cm in length and of grayish-white appearance, were successfully obtained. The specimens were processed for histopathological analysis.

**Figure 2:**
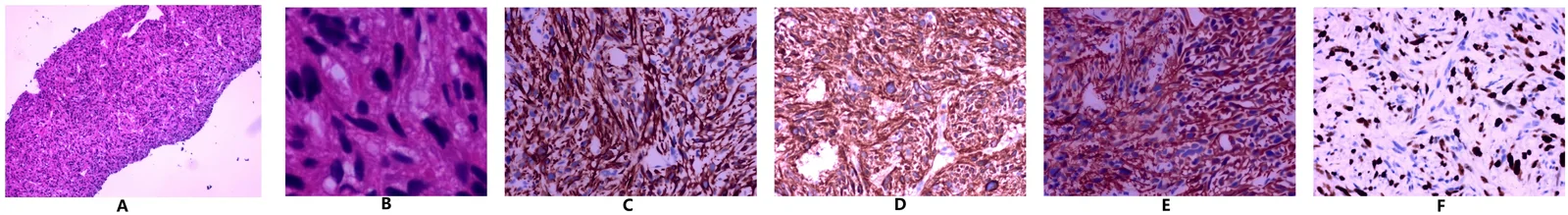
Histopathological and immunohistochemical features. **(A)** Low-power hematoxylin and eosin (H&E) staining (×100) demonstrates the overall histological architecture and fascicular growth pattern of the tumor tissue. **(B)** Hematoxylin and eosin (H&E) staining (×400) shows malignant spindle cells arranged in interlacing fascicles with nuclear atypia. Immunohistochemical staining reveals positivity for **(C)** desmin (diffuse cytoplasmic), **(D)** smooth muscle actin (SMA) and **(E)** caldesmon, confirming smooth muscle origin. **(F)** Ki-67 proliferation index is approximately 70%. The tumor is negative for HMB45, CD34, S-100, p53, myogenin, hepatocyte, and Epstein-Barr virus-encoded RNA (EBER) (not shown). EMA shows focal positivity (not shown), and p16 demonstrates diffuse nuclear expression (not shown). These findings are consistent with intermediate-grade (FNCLCC grade 2) leiomyosarcoma.

Microscopic evaluation of the biopsy specimens revealed a mesenchymal-derived malignant tumor. Histologically, moderately atypical spindle cells in interlacing fascicles were observed, with 10–19 mitoses/10 HPF and <50% necrosis. The tumor was FNCLCC grade 2 (intermediate) with a total score of 5 (differentiation 2, mitoses 2, necrosis 1).Immunohistochemical staining showed positive expression of Desmin, smooth muscle actin (SMA), Caldesmon, p16 (diffusely positve), and epithelial membrane antigen (EMA, focally positive); negative expression of HMB45, CD34, S-100, p53, Myogenin (negative in sarcoplasm), and Hepatocytes.The proliferation index, as assessed by Ki-67 immunohistochemistry, was approximately 70%. *In situ* hybridization for Epstein-Barr virus-encoded RNA (EBER) was negative. This immunohistochemical panel allowed exclusion of other spindle cell neoplasms, including peripheral nerve sheath tumors (S-100 negative), solitary fibrous tumor (CD34 negative), and perivascular epithelioid cell tumor (HMB45 negative). Epithelial malignancies were also largely excluded due to the absence of diffuse epithelial marker expression. The consistent positivity for smooth muscle markers confirmed smooth muscle differentiation, establishing the diagnosis of leiomyosarcoma. (**Figure 2**).

**Figure 3:**
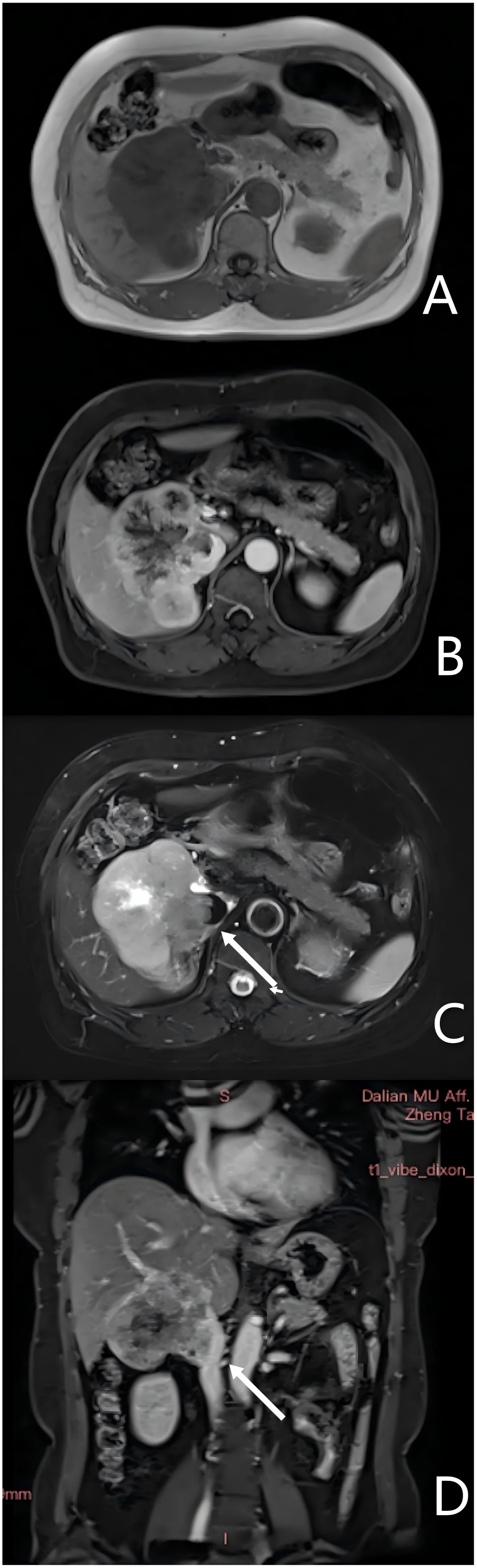
Magnetic resonance imaging (MRI) findings. **(A)** Axial T1-weighted image shows a hypointense mass in the right hepatic region. **(B)** Axial contrast-enhanced T1-weighted image demonstrates heterogeneous enhancement. **(C)** Axial T2-weighted image reveals heterogeneous hyperintensity within the mass, with evidence of tumor involvement within the IVC lumen (arrow). **(D)** Coronal contrast-enhanced T1-weighted multiplanar reconstruction (MPR) shows tumor continuity with the IVC lumen (arrow), indicating IVC origin.

To determine the origin, imaging was re-evaluated. MRI review confirmed tumor within the IVC on T2-weighted images (**Figure 3**). Concurrently, a non-blinded retrospective review of the stored ultrasound cine loops was performed by a senior ultrasound physician with 20 years of experience in clinical ultrasonography. The review identified a hypoechoic component within the IVC lumen that was continuous with the hepatic mass and had not been recognized during the initial examination (**Figure 1E**). In addition, luminal narrowing and increased blood flow velocity were observed in the IVC, which we initially interpreted as secondary to external compression of the IVC by the tumor, overlooking the presence of an intraluminal hypoechoic lesion (**Figure 1F**). The corresponding dynamic ultrasound clips are available in the **Supplementary Material**. No other primary site was found. The final diagnosis was primary IVC LMS with hepatic and pulmonary metastases. Given metastatic disease, the patient opted for systemic chemotherapy over surgery and received treatment at an external hospital. Telephone follow-up at 6 months after diagnosis confirmed that the patient was still undergoing chemotherapy; however, the patient was lost to further follow-up thereafter, and long-term clinical outcome data are unavailable. A detailed timeline of the patient's clinical presentation, investigations and management is summarized in **Table 1**.

**Table 1:** Timeline of clinical presentation, investigations, and management.

Time point	Clinical event/symptom	Diagnostic procedure/imaging	Key findings/diagnosis	Management/outcome
2 years before admission	Persistent dull right upper quadrant (RUQ) pain	None documented	Symptomatic onset	Not applicable
2 months before admission	Worsening RUQ pain radiating to back; abdominal distension and diarrhea	Initial abdominal CT (external hospital)	Hypodense mass in right liver lobe; suspected liver tumor	Referred for further evaluation
At admission to our hospital	Same symptoms	Laboratory tests	Elevated NSE (25.9 ng/mL); normal other tumor markers and viral serology	Admission for workup
Day 1	–	Abdominal ultrasound (grayscale + Doppler)	10.6 × 7.8 cm hypoechoic mass in right lobe with heterogeneous echotexture, multiple strip-like hyperechoic areas, and anechoic regions; abundant vascularity	Suspicion of primary hepatic malignancy, possibly sarcoma, specifically hepatic leiomyosarcoma
Day 1	–	Chest CT	Multiple bilateral pulmonary nodules	Suspicion of metastatic disease
Day 3	–	Abdominal MRI	Heterogeneous mass with T2 hyperintensity, diffusion restriction, heterogeneous enhancement; involvement of pancreatic head and right adrenal suspected	Malignant tumor confirmed; IVC involvement not initially noted
Day 4	–	Contrast-enhanced ultrasound (CEUS)	Peripheral-to-central arterial enhancement, iso-enhancement in portal phase, washout in venous phase, central necrosis (malignant enhancement pattern)	Supports malignant nature
Day 4	–	Ultrasound-guided percutaneous biopsy (16G needle)	Two tissue cores obtained	Specimen sent for histopathology
Day 7	–	Histopathology and immunohistochemistry report	Desmin (+), SMA (+), Caldesmon (+), Ki-67 ~70%; consistent with leiomyosarcoma	Pathological diagnosis of leiomyosarcoma established
Day 8	–	Multidisciplinary imaging review (MRI + ultrasound)	Retrospective identification of intraluminal IVC component; tumor origin reassessed	Final diagnosis: Primary IVC leiomyosarcoma with hepatic and pulmonary metastases
After diagnosis	Stable clinical condition	Treatment planning discussion	Patient declined surgical resection due to metastatic disease	Referred for systemic chemotherapy at another institution
Six-month follow-up	Under chemotherapy	Regular imaging surveillance	Condition stable under treatment	Palliative chemotherapy ongoing

## Discussion

IVC LMS carries a poor prognosis, with complete surgical resection being only potentially curative treatment (3). Key predictors of survival include negative resection margins, smaller tumor size, and successful en bloc radical resection (6, 7). Larger tumor size at detection often precludes complete resection (19). Early and accurate diagnosis is critical.

Imaging plays a vital role in the management of IVC LMS (8). CT often serves as the preferred initial imaging modality and MRI offers superior soft-tissue characterization, particularly for assessing intraluminal components (5). Unfortunately, contrast-enhanced CT was not performed initially, limiting identification of the tumor’s IVC origin. Initial MRI detected a malignant tumor but did not suggest IVC origin or leiomyosarcoma. This highlights the diagnostic challenge radiologists face with this rare entity due to inter-observer variability and limited prior experience. Several factors contributed to this initial oversight: the rarity of IVC leiomyosarcoma biased initial interpretation toward common primary hepatic neoplasms rather than a vascular wall origin, while the tumor’s predominantly extraluminal growth pattern—with most of its volume within the hepatic parenchyma—masked imaging signs of IVC origin and hindered prospective diagnosis. The intraluminal component and tumor–IVC continuity were only clearly identified on targeted retrospective review by more experienced radiologists, prompted by the histological diagnosis of leiomyosarcoma and the known rarity of primary hepatic leiomyosarcoma, underscoring the importance of actively assessing the relationship between large abdominal masses and adjacent major vessels.The role of ultrasound in evaluating IVC LMS is less documented. Some studies suggest ultrasound can serve as an initial examination, typically helping determine its precise anatomical relationship with the IVC (9). Xu et al. (4) have also reported that IVC LMS often appears on ultrasound as a lobulated, hypoechoic mass causing IVC obstruction. Differential diagnoses of intraluminal leiomyosarcomas are scarce, a tumor within the IVC with expansion of its lumen is highly suggestive of an IVC LMS. Extraluminal leiomyosarcomas which can invade surrounding tissues, must be differentiated from other retroperitoneal or peripheral organ tumors (9). Establishing the relationship between mass and the IVC is therefore crucial.

Qian XJ et al. highlighted key imaging clues for IVC leiomyosarcoma, including IVC dilation, an imperceptible IVC lumen at maximal tumor contact, and collateral vein formation (10). Webb EM et al. (11) identified CT signs predictive of IVC origin: the “positive embedded sign,” “negative embedded sign,” and “imperceptible IVC sign.” The imperceptible IVC sign is highly predictive of IVC origin; the negative embedded sign helps exclude it. A positive embedded sign—where an organ is embedded in the periphery of a larger mass—suggests the mass originates from that organ (12). In this case, dynamic ultrasound demonstrated the mass embedded within and narrowing the IVC lumen, analogous to the “positive embedded organ” sign, suggesting its primary IVC origin. Further progression could have manifested the “imperceptible IVC sign.”

Prone imaging enhances diagnostic accuracy by demonstrating persistent tumor contact with the IVC wall or non-visualization of the lumen—key indicators of IVC origin in predominantly extraluminal lesions (13). This method aligns well with ultrasound’s capability for dynamic scanning with positional changes. Studies confirm that imperceptible IVC lumen at maximal tumor contact and persistent contact in prone position are strong predictors of IVC origin (14). Therefore, employing positional changes during ultrasound examination is a feasible and valuable method for assessing the tumor-IVC relationship.

Intravascular ultrasonography (IVUS) can provide high-resolution visualization of the IVC wall, aiding in distinguishing between extrinsic compression and true tumor invasion (15). However, its invasive nature and high cost restrict its application to cases where non-invasive imaging fails to adequately define IVC involvement. Additionally, distinguishing hypoechoic tumor margins from the vessel lumen may remain challenging with IVUS.

Ultrasound is valuable for assessing the tumor-IVC relationship. An intraluminal mass raises suspicion of IVC origin—especially leiomyosarcoma, the most common primary IVC malignancy—making ultrasound a useful tool for suggesting IVC LMS. Ultrasound in this case demonstrated an intraluminal mass within the IVC with extra-luminal hepatic extension. Pathologically, it can be challenging to differentiate primary IVC LMS from secondary invasion (16). Given the rarity of primary hepatic leiomyosarcoma and the known metastatic propensity of IVC LMS to the liver and lungs (5, 7, 17–19), a primary IVC origin with hepatic metastasis was considered most plausible.

Beyond the commonly reported features (large size, heterogeneous echogenicity), this case exhibited multiple strip-like hyperechoic regions—a finding also noted in other IVC LMS reports (4, 5, 20) and in leiomyosarcomas of peripheral veins (21, 22). This pattern may correlate with intratumoral stromal fibrosis (23), and we propose it as a distinctive ultrasonographic feature of IVC LMS. Notedly, the presence of these hyperechoic multiple stripes prompted consideration of a smooth muscle tumor, drawing an analogy to the palisading hyperechoic stripes seen in uterine leiomyomas. This parallel is clinically relevant: IVC LMS occurs predominantly in women and has been associated with uterine disease (24). While uterine leiomyosarcomas are rare and can sonographically mimic benign leiomyomas (25), key malignant features—large size, heterogeneous echotexture, irregular anechoic areas, absence of acoustic shadowing, and increased vascularity (26, 27) — are well-established in gynecologic ultrasound. Leveraging this imaging overlap between uterine and vascular smooth muscle tumors allows sonographers to apply experience gained from uterine pathology to enhance diagnostic confidence for leiomyosarcomas in extra-uterine sites, including the IVC. In this case, the initial ultrasound physician suspected leiomyosarcoma because the mass sonographically resembled a uterine smooth muscle tumor. However, limited familiarity with IVC leiomyosarcoma and the predominantly intrahepatic location of the mass resulted in inadequate assessment of the tumor–IVC relationship and misjudgment of the tumor’s site of origin.

On targeted retrospective review after histopathological confirmation of leiomyosarcoma, a senior ultrasound physician identified the intraluminal component of the tumor within the IVC. This review was not blinded, and prior knowledge of the histopathological diagnosis may have prompted a more focused reassessment of the tumor–IVC relationship. Differences in the physicians’ levels of experience may also have contributed to the discrepancy between the initial and retrospective interpretations. Nevertheless, this case illustrates the potential value of ultrasound in evaluating the relationship between a mass and the IVC. In particular, identification of a tumor component within the IVC lumen that is continuous with the extraluminal mass may raise suspicion of an IVC origin. Other supportive, although nonspecific, sonographic findings include heterogeneous echotexture, irregular anechoic areas, and strip-like hyperechoic regions. These observations require validation in larger case series or multicenter studies because the rarity of IVC leiomyosarcoma limits systematic investigation, and the available evidence remains largely case-based.

Although each rare tumor type affects few patients, rare cancers collectively impose a substantial health burden, accounting for approximately 22% of all cancer diagnoses. No universal definition exists; criteria vary significantly by region and research context. While the U.S. Orphan Drug Designation Program and the European Union define rare diseases based on prevalence (e.g., <64/100,000 and <5/10,000, respectively), oncology research increasingly favors incidence-based thresholds. The RARECARE project defines rare cancers as those with an incidence <6/100,000 per year, a threshold further refined to <2/100,000 by the International Rare Cancers Initiative (IRCI) to facilitate clinical trial development (28).These challenges underscore the necessity of multicenter collaboration and data sharing to establish a robust evidence base for rare tumors like IVC leiomyosarcoma.

This limitation of small datasets for rare diseases also hinders the development of computer-aided diagnosis models. Deep learning models, particularly convolutional neural networks (CNNs), learn imaging patterns directly from examples but generally require large, carefully annotated datasets when trained from scratch. Transfer learning may reduce this requirement by adapting a model previously trained on a larger dataset to a new task with fewer cases. Cross-organ transfer learning applies knowledge learned from images of one organ to another, whereas cross-modality transfer learning adapts knowledge across imaging techniques, such as from CT or MRI to ultrasound. Lee and Nishikawa demonstrated the feasibility of an intermediate medical-imaging transfer strategy across different organs and modalities (29). Such approaches could potentially facilitate model development for rare tumors such as IVC leiomyosarcoma by drawing on larger related imaging datasets. However, this remains a research direction requiring multicenter data sharing, standardized annotation, external validation, and evaluation across institutions and imaging systems.

## Conclusion

While the key finding of intraluminal extension was made on targeted retrospective ultrasound re-evaluation, this case analysis still underscores the utility of ultrasound in evaluating the tumor-IVC relationship, a key determinant in diagnosing IVC LMS. Sonographic features such as a heterogeneous hypoechoic mass with irregular anechoic areas and, notably, multiple strip-like hyperechoic regions, can provide valuable diagnostic clues. Moreover, adopting a cross-organ diagnostic approach, informed by the sonographic characteristics of smooth muscle tumors in other locations, may further enhance diagnostic confidence. Ultimately, advancing this concept through cross-organ, cross-modality analytical frameworks may offer a promising solution to improve the diagnosis and understanding of rare tumors like IVC LMS.

## Data Availability

The original contributions presented in the study are included in the article/**Supplementary Material**. Further inquiries can be directed to the corresponding author.
